# Immune Imbalances in Non-Alcoholic Fatty Liver Disease: From General Biomarkers and Neutrophils to Interleukin-17 Axis Activation and New Therapeutic Targets

**DOI:** 10.3389/fimmu.2016.00490

**Published:** 2016-11-11

**Authors:** Feliciano Chanana Paquissi

**Affiliations:** ^1^Department of Medicine, Clínica Girassol, Luanda, Angola

**Keywords:** non-alcoholic fatty liver disease, non-alcoholic steatohepatitis, neutrophil-to-lymphocyte ratio, Th17 cells, Treg cells, interleukin-17

## Abstract

Non-alcoholic fatty liver disease (NAFLD) is an increasing problem worldwide and is associated with negative outcomes such as cirrhosis, hepatocellular carcinoma, insulin resistance, diabetes, and cardiovascular events. Current evidence shows that the immune response has an important participation driving the initiation, maintenance, and progression of the disease. So, various immune imbalances, from cellular to cytokines levels, have been studied, either for better compression of the disease pathophysiology or as biomarkers for severity assessment and outcome prediction. In this article, we performed a thorough review of studies that evaluated the role of inflammatory/immune imbalances in the NAFLD. At the cellular level, we gave special focus on the imbalance between neutrophils and lymphocytes counts (the neutrophil-to-lymphocyte ratio), and that which occurs between T helper 17 (Th17) and regulatory T cells as emerging biomarkers. By extension, we reviewed the reflection of these imbalances at the molecular level through pro-inflammatory cytokines including those involved in Th17 differentiation (IL-6, IL-21, IL-23, and transforming growth factor-beta), and those released by Th17 cells (IL-17A, IL-17F, IL-21, and IL-22). We gave particular attention to the role of IL-17, either produced by Th17 cells or neutrophils, in fibrogenesis and steatohepatitis. Finally, we reviewed the potential of these pathways as new therapeutic targets in NAFLD.

## Introduction

Non-alcoholic fatty liver disease (NAFLD) is a health problem with increasing prevalence worldwide (1, 2), mainly due to the increased rates of obesity and type 2 diabetes (3, 4), with increasingly installation at an early age (4, 5). The prevalence of NAFLD in the general adult population is around 25.24% (6), reaching 67.5 and 74% among those with obesity or diabetes, respectively (7), with 10–12.2% of them having subclinical hepatic inflammation, known as non-alcoholic steatohepatitis (NASH), and/or fibrosis (8). NAFLD is associated with significant morbidity and mortality, increasing the risk of cirrhosis, hepatocellular carcinoma (HCC), insulin resistance, metabolic syndrome, diabetes, cardiovascular events, and cardiovascular and liver disease mortality (2, 9–13), being, therefore, recognized as a multisystem disease (14).

Recent investigations have highlighted the function of the immune response as a driver in the initiation, maintenance, and progression of NAFLD (15–17). General inflammatory/immunity biomarkers, as C-reactive protein (CRP), interleukins, have been associated with the occurrence and prognosis of NAFLD, including the link to vascular events (15, 18–21). In the cellular level, various immune imbalances have also emerged as biomarkers in NAFLD, from those in general white blood cells such as neutrophil-to-lymphocyte ratio (NLR), to specific lymphocytes subsets (22–24). The NLR expresses an imbalance in leukocytes with the dominance of neutrophils over lymphocytes and has been increasingly recognized as a predictor of outcomes in NAFLD (25), a role also shown in other chronic liver diseases such as viral hepatitis, liver cirrhosis, and HCC (26–28). Another described cellular imbalance is what occurs at the CD4^+^ cells level with the dominance of T helper 17 (Th17) subset over the regulatory T (Treg) cells, which results from the polarization of the differentiation of T helper cells, also present in NAFLD and other chronic liver diseases (24, 29–32). The functional equilibrium between Th17 and Treg in peripheral blood is an important element to ensure the equilibrium between the defense and the autoimmunity; and there is an interplay and plasticity between these cells and their subsets, with cellular polarizations and different cytokine profiles in the presence of different stimuli (33–36). The cellular imbalances reflect at the molecular level through pro-inflammatory cytokines including those involved in Th17 differentiation [IL-6, IL-21, IL-23, and transforming growth factor-beta (TGF-β)] (33, 37–39), and those released by Th17 cells (IL-17A, IL-17F, IL-21, and IL-22) (29, 40–42).

In this article, we performed a thorough review of studies that evaluated the role of inflammatory/immune imbalances in the pathophysiology and outcome prediction in NAFLD. We focused the neutrophil/lymphocyte and Th17/Treg imbalances as emerging biomarkers at the cellular level; its reflection at the molecular level through pro-inflammatory cytokines with particular attention to the role of IL-17, either produced by Th17 cells or neutrophils, in fibrogenesis and steatohepatitis. Finally, we reviewed the potential of these pathways as new therapeutic targets in NAFLD.

## An Overview of the Predictive Role of General Inflammatory Biomarkers in NAFLD

Several inflammatory markers such as CRP and cytokines have been associated with NAFLD (15, 16, 20, 43). In a study with individuals without obesity, the presence of hs-CRP, with or without insulin resistance, was associated with higher prevalence of NAFLD, with a significant risk increase as increased the value of hs-CRP [odds ratio (OR) de 1.37; 95% confidence interval (CI): 1.06–1.77] for each 1 SD above (1.48 mg/L) (16). In another study, a positive association between the degree of steatosis and hs-CRP was observed (*P* < 0.05) after adjusting for BMI (44). Additionally, an elevated CRP is a predictor of disease progression and severity, as shown that hs-CRP was significantly higher in cases of NASH than in simple steatosis (19, 45); furthermore, among patients with NASH, hs-CRP was significantly elevated in those with advanced fibrosis compared with those with mild, even after adjustment for confounders (19).

Other inflammatory biomarkers associated with NAFLD and its progression are cytokines. The expression of interleukin-6 (IL-6) is markedly increased in the liver cells of patients with simple steatosis (*P* < 0.005) or NASH (*P* < 0.010) compared to normal subjects (46). IL-6 expressed in hepatocytes, and its level in the blood, correlates positively with the degree of liver inflammation, and fibrosis (46). Another key cytokine in NAFLD is interleukin-17 (IL-17) (41). The activation of the IL-17 axis has shown to have a central role in the progression from NAFLD to NASH in experimental studies (15, 24, 41, 42). The role of IL-17 family cytokines will be discussed further elsewhere in this paper. Similarly, TGF-β, a cytokine known by its fibrotic effect in many organs is involved in the progression of NAFLD (47, 48). In a study that evaluated the gene expression of this cytokine in NAFLD, there was a marked increase in TGF-β1 gene expression in patients with NASH compared with simple steatosis (*P* = 0.0002) (49). In another study with 1,322 healthy subjects without other risk factors, serum TGF-β3 levels was significantly higher in those who developed NAFLD than in those who did not (mean 554 vs. 285 pg/ml; *P* = 0.002) after 4 years of follow-up; and as the TGF-β3 tertiles increased, there was a significant increase in NAFLD incidence (6.3, 38.0, and 55.7% for the first, second, and third tertiles, respectively; *P* < 0.05) (50).

The inflammatory response seems to be an important mediator of many NAFLD-associated outcomes such as the HCC by creating an inflammatory microenvironment (28, 51), cardiovascular disease by promoting atherogenesis (52, 53). Table 1 summarizes the clinical studies that have assessed the role of general inflammatory biomarkers and cytokines to predict outcomes in NAFLD.

**Table 1:** **Clinical studies that have assessed the role of general inflammatory biomarkers and cytokines to predict outcomes in NAFLD**.

Reference	Biomarker	Number of patients	Results
Park et al. (16)	CRP	120 non-obese patients with NAFLD and 240 matched controls	Multivariate analysis showed that CRP (OR = 1.37; 95% CI 1.06–1.77 per 1 SD increase) and HOMA-IR [OR = 2.28; 95% CI: 1.67–3.11, per 1 SD (0.63)] were independent risk factors for NAFLD in non-obese patients

Yoneda et al. (19)	Hs-CRP and CRP mRNA	100 patients with NAFLD (29 with steatosis and 71 with NASH)	Patients with NASH had significantly elevated serum hs-CRP (*P* < 0.0048) and increased intrahepatic expression of the CRP mRNA (*P* = 0.0228) than those with simple steatosis. In addition, patients with advanced fibrosis stages (F3–4) had a significantly higher serum hs-CRP than those with mild (F1–2) (*P* < 0.0384), even after adjustment for confounders

Oruc et al. (54)	CRP	50 NAFLD cases and 50 healthy controls	Serum CRP levels were significantly higher in simple steatosis and NASH groups compared to healthy controls (mean: 7.5 and 5.2 vs. 2.9 mg/dl, respectively, *P* < 0.01)

Riquelme et al. (18)	Hs-CRP	832 Hispanic subjects who underwent abdominal ultrasound	The prevalence of NAFLD was 23%. A high hs-CRP (>0.86 mg/L) was associated with NAFLD in multivariate analysis (OR 2.9; 95% CI 1.6–5.2); as was a high body mass index, abnormal aspartate aminotransferase, and insulin resistance

Zimmermann et al. (44)	Hs-CRP	627 obese adults	A positive association between degree of steatosis and hs-CRP was observed (*P* < 0.05), and this effect remained significant after adjusting for BMI, lobular inflammation, hepatocyte ballooning, and fibrosis

Wang et al. (55)	Hs-CRP	8,618 initially NAFLD-free Chinese subjects who underwent annual health screen	The hs-CRP level was independently associated with NAFLD. The incidence ratio of NAFLD increased significantly with increasing hs-CRP quartiles either in man (21.1, 18.6, 24.8, and 31.1% for the first, second, third, and fourth quartiles, respectively), and in females (6.2, 6, 11.4, and 19.5% for the first, second, third, and fourth quartiles, respectively). The association was stronger in females than in males

Cayón et al. (49)	TGF-β1 and leptin systems	90 subjects with NAFLD (55 with NASH and 35 with simple steatosis)	There was a marked increase in intrahepatic gene expression of TGF-β1 (*P* = 0.0002), leptin receptor mRNA (*P* = 0.0016), and its protein (*P* < 0.05) in patients with NASH. A strong correlation was shown between leptin receptor gene expression and TGF-β1 gene expression (*P* = 0.023)

Wei et al. (50)	TGF-β3	1,322 healthy subjects without other risk factors, followed during 4 years	After 4 years of follow-up, the cumulative incidence of NAFLD was 25.3% (334/1,322). Those who developed NAFLD had higher serum TGF-β3 levels than those who did not (mean 554 vs. 285 pg/ml; *P* < 0.002); and the incidence increased significantly with increasing TGF-β3 tertiles (6.3, 38.0, and 55.7%, for the first, second, and third tertiles, respectively; *P* < 0.05)

Wieckowska et al. (46)	IL-6 and IL-6 mRNA	50 patients with suspected NAFLD	IL-6 mRNA expression was markedly increased in the livers of patients with NASH than in those with simple steatosis (*P* < 0.005) or normal biopsies (*P* < 0.010). There was a positive correlation between hepatocyte IL-6 mRNA expression and degree of inflammation, stage of fibrosis, plasma IL-6 levels, and degree of systemic insulin resistance

Bahcecioglu et al. (56)	TNF-α and IL-8	42 patients (28 with NASH and 14 with cirrhosis) and 15 healthy controls	Serum TNF-α levels were significantly higher in patients with NASH and cirrhosis than in healthy controls (*P* < 0.05). Serum IL-8 levels in patients with NASH (*P* < 0.001) and cirrhosis (*P* < 0.05) were significantly higher than in the healthy control group

Coulon et al. (43)	TNF-α, IL-6, and TNF-α mRNA	92 subjects (30 obese with steatosis, 32 with NASH, and 30 healthy controls)	In comparison with controls, serum IL-6 was significantly high both in simple steatosis (mean 2.863 vs. 1.224 pg/ml; *P* < 0.001) and NASH patients (mean 3.136 vs. 1.224 pg/ml; *P* < 0.001), whereas serum TNF-α elevation was only significant in NASH group (mean 1.803 vs. 1.405 pg/ml; *P* = 0.026). Patients with NASH had a significantly higher expression of TNF-α mRNA in liver tissue than those with simple steatosis

Seo et al. (57)	TNF-α	363 apparently healthy subjects	At 4 years of follow-up, the cumulative incidence of NAFLD was 29.2% (106/363). Those who developed NAFLD had higher serum TNF-α levels than those who did not (mean 3.65 vs. 3.15 pg/ml; *P* < 0.01). The incidence of NAFLD increased significantly with increasing TNF-α tertiles (22.6, 35.8, and 41.5%, for the first, second, and third tertiles, respectively; *P* < 0.05). The risk of developing NAFLD was significantly higher in the highest tertile of TNF-α than in the lowest (OR, 2.20; *P* < 0.05)

Paredes-Turrubiarte et al. (58)	TNF-α and IL-10	102 morbidly obese	Patients with NAFLD showed increased TNF-α than those with morbidly obese subjects but without NAFLD (mean 37.41 vs.31.41 pg/ml, *P* < 0.046). Serum levels of IL-10, in contrast, were decreased in NAFLD (mean 61.05 vs. 76.40 pg/ml, *P* < 0.002), which suggests an imbalance between the pro-inflammatory and anti-inflammatory cytokines

Tang et al. (29)	IL-17, IL-21, and IL-23	58 human liver specimens (14 with NASH and 40 controls^b^)^a^	There was a significant increase of IL-17(+) cells infiltrating the liver of NASH patient and increased gene expression of Th17 cell-related cytokines (IL-17, IL-21, and IL-23). Hepatic Th17 cells and IL-17 were associated with steatosis and pro-inflammatory response in NAFLD and facilitated the transition from simple steatosis to steatohepatitis

Okumura et al. (59)	LECT2	231 Japanese adult tested for LECT2	Serum LECT2 was significantly high in patients with fatty liver than in those without (mean 48.7 vs. 140.5 ng/ml; *P* < 0.001)

*HOMA-IR, homeostasis model assessment-insulin resistance; IL-10, interleukin-10; IL-17, interleukin-17; IL-21, interleukin-21; IL-23, interleukin-23; IL-6, interleukin-6; mRNA, messenger RNA; NASH, non-alcoholic steatohepatitis; LECT2, leukocyte cell-derived chemotaxin 2; TNF-α, tumor necrosis factor-alpha*.

*^a^The study included animal experiments*.

*^b^Control specimens obtained from the liver tissues besides resected hemangiomas*.

## The Role of Cellular Immune Imbalances in NAFLD

In the last 5 years, many cellular imbalances in the immune response have been associated with NAFLD and its prognosis (23, 25), which have brought a cellular background to what was observed through pro-inflammatory cytokines. These cellular imbalances range from the simple increase in the total count of leukocytes (23) to specific lymphocytes subpopulations (25). This was remarkable in a study with 3,681 healthy subjects in which, as increased the WBC count quartile above the lowest, there was a significant increase in NAFLD risk in both men [OR (95% CIs): 1.48 (1.10–1.98), 1.59 (1.18–2.14), and 1.84 (1.35–2.51) for the second, third, and fourth quartiles, respectively]; and women [OR (95% CIs): 1.15 (0.67–1.96) 1.88 (1.13–3.11), and 2.74 (1.68–4.46) for the second, third, and fourth quartiles, respectively] (23). Among these imbalances, we highlight that which occurs at the level of T helper cellular subsets (Th17/Treg imbalance), and that between neutrophils and lymphocytes counts (expressed by NLR).

### The Th17/Treg Imbalance

An important cellular imbalance that has emerged as a cellular background in the role of the inflammatory response in NAFLD is the Th17/Treg imbalance. In a study with 94 subjects (30 with NASH, 31 with NAFLD, and 43 healthy controls), patients with NASH (and in less degree with steatosis) had a lower frequency of T regulatory cells in peripheral blood, in comparison with controls (30). In addition, the progression from steatosis to NASH was marked by a higher frequency of Th17 cells in the liver and an increased Th17/resting T regulatory cell ratio in the liver and in peripheral blood (30).

In experimental models, liver infiltration by Th17 cells showed to be a critical element for NASH initiation and development of fibrosis in wild-type mice, and this infiltration was accompanied by an increase in the production of pro-inflammatory cytokines (IL-6, TNF-α, and TGF-β) (42). In another study using mice fed with high-fat diet (HFD), there was a significant increase of Th17 cells in the liver (*P* < 0.02) and the abdominal adipose tissue (AAT) (*P* < 0.01), without a concurrent increase of Treg (60). NASH and metabolic alterations occurred in mice-fed HFD, and Th17 cells (either AAT or liver-derived) positively correlated with NASH (60). Other studies have shown in parallel that the reduction, dysfunction, or disproportionate number of Treg cells contributes to the progression to NASH because Treg cells play a critical role in regulating the inflammatory processes in the liver (24, 29, 30). This cellular imbalance is accompanied by the activation of the IL-17 axis, and an increase of other pro-inflammatory cytokines such as IL-6, and TNF-α (42, 58); and its value has been highlighted by the demonstration that therapies targeted to reverse this imbalance have shown the potential to alleviate steatosis and the progression to NASH (32, 61, 62).

#### Lymphocytes Site-Specific Source

An important element to consider is the site specificity of lymphocytes in NAFLD, as shown in an experimental study where it was observed that the cells infiltrating the liver were labeled lymphocytes that migrated predominantly from mesenteric lymph nodes (MLN) than from spleen, bone marrow, or thymus (63), suggesting that the gut is the primary source of cellular elements involved in NAFLD pathogenesis, which is in turn affected by the microbiota (64, 65).

### Neutrophil-to-Lymphocyte Ratio as a Cellular Biomarker in NAFLD

Neutrophil-to-lymphocyte ratio is a derivative biomarker obtained from the absolute counts of neutrophils and lymphocytes. NLR is a cellular imbalance (with the dominance of neutrophils over the lymphocytes) that has been found to be related to a lot of diseases that share the chronic inflammatory response as critical in pathogenesis, such as cancer and cardiovascular diseases (66, 67). In a nationally representative American sample, including 9,427 subjects, the average NLR was 2.15 in the general population, and values above these were found in those with chronic inflammatory states including cardiovascular diseases and diabetes (68). The potential of NLR as a biomarker has also been shown in liver diseases, which reflects the pathologic effects of the dominance of activated neutrophils, an important effector cell of the innate immunity, in diseases of this organ (69, 70).

#### NLR and NAFLD Severity

In NAFLD, the NLR is associated with high disease severity, as found in a study with 101 patients where NASH patients had higher NLR compared with those without (mean 2.5 vs. 1.6, *P* < 0.001) (25). NLR showed a good correlation with the NAFLD activity score and its individual components (steatosis, inflammation, and ballooning *P* < 0.001), and advanced fibrosis stages (F3–4) compared with lower stages (F1–2) (median 2.9 vs. 1.8, respectively, *P* < 0.001) (25). In another study including 873 patients with biopsy-proven NAFLD (and 150 healthy controls), NLR was higher in NASH patients than in non-NASH cases (mean 2.6 vs. 1.9, respectively, *P* < 0.001); and similarly, patients with advanced fibrosis stages (F3–4) had a higher NLR compared with those in early (F1–2) (mean 2.5 vs.1.8, respectively, *P* < 0.001) (71). In study comparing the role of this biomarker in three liver diseases (NASH, HBV, and HCV hepatitis), NLR was significantly higher in NASH patients compared to HBV, and HCV, or controls (*P* < 0.001, *P* < 0.001, and *P* < 0.001, respectively) (22), suggesting a higher contribution of this imbalance in NASH than in other chronic liver diseases.

#### NLR and NAFLD Prognostisis

Besides being a marker of disease severity, NLR is also a predictor of mortality (72). In a study including 570 patients with end-stage cirrhosis (including 54 due to NAFLD) listed for liver transplantation, NLR ≥ 5 was associated with higher 3-month mortality (OR 6.02, *P* < 0.043); and as increased NLR, there was a significant increase in proportion of patients who died within 3 months of listing (3, 13.8, and 37.3%, for NLR < 2, 2–4.9, and ≥5, respectively, *P* < 0.001) (72). NLR is also a predictor of higher NAFLD score, advanced fibrosis, and severe ascites (25, 71, 72).

#### NLR in NAFLD-Associated Conditions

The role of NLR seems to begin long before and extend beyond the NAFLD. This is evident because subjects with obesity and diabetes, which are the main risk factors for NAFLD, show higher average NLR in relation to controls (68, 73), and among patients with morbid obesity, the mean NLR values were significantly higher in those who developed type 2 diabetes (T2DM) than those who did not (mean 4.11 vs. 3.46, *P* < 0.001) (73), showing that a higher inflammatory states precedes both (NAFLD and T2DM) obesity-related outcomes.

A high NLR remains an independent predictor of poorer outcome even in those that evolved to terminal stages of chronic liver disease – cirrhosis and/or HCC (27, 74, 75). In the evolution of patients with HCC undergoing radiofrequency ablation, or surgical resection, a high NLR predicted poor outcomes with higher recurrence and mortality rates (74–77) and poor overall survival even after a curative liver resection (76). And even in those undergoing liver transplantation, a high NLR was associated with poorer overall and recurrence-free survival (78).

#### Neutrophils as Important Source of IL-17 in Liver Diseases

Recent investigations have found that neutrophils are themselves an important source of IL-17 in the human liver, especially in late fibrosis stages (56, 70). Therefore, these researches come to bring a pathophysiological background to the predictive role of elevated NLR in this disease, while it occurs with an important IL-17 axis activation, besides other possible mechanisms beyond our understanding. This role of neutrophils as a source of IL-17 has just been found in other organic diseases such as the kidney (79) and airways (80).

### Other Cellular Imbalances in NAFLD

Other immune system cells that have been found imbalanced in frequency and that appear to be involved in the cross talk with hepatocytes, hepatocellular damage, and in the transition from NASH to HCC are natural killer T cells and CD8^+^ lymphocytes (81, 82). Table 2 summarizes the clinical studies that evaluated the role of cellular imbalances as drivers and predictors of outcomes in NAFLD.

**Table 2:** **Clinical studies on the value of cellular immune imbalances as drivers and predictors of outcomes in NAFLD**.

Reference	Cellular biomarker	Number of patients	Results
Lee et al. (23)	WBC	3,681 healthy subjects who underwent medical checkup	The risk of NAFLD increased significantly as WBC increased. Compared with the lowest WBC count quartile, the respective ORs (95% CIs) for the second, third, and fourth quartiles were 1.48 (1.10–1.98), 1.59 (1.18–2.14), and 1.84 (1.35–2.51) for men; and 1.15 (0.67–1.96), 1.88 (1.13–3.11), and 2.74 (1.68–4.46) for women

Wang et al. (83)	WBC count	15,201 participants without NAFLD who underwent health checkups between 2005 and 2011	There were 3,376 new cases of NAFLD, and WBC count was a predictor of its incidence. Compared with the lowest WBC quartile (Q1), the HRs (95% CIs) were 1.09 (0.97–1.21), 1.17 (1.05–1.30), and 1.15 (1.03–1.28) for Q2, Q3, and Q4 quartiles, respectively, after adjusting for potential confounders

Alkhouri et al. (25)	NLR	101 patients with suspected NAFLD who underwent liver biopsy	Patients with NASH had a higher NLR than those without (median 2.5 vs. 1.6, *P* < 0.001). The NLR correlated with the NAFLD activity score and its individual components (steatosis, inflammation, and ballooning *P* < 0.001). Patients with advanced fibrosis (F3–4) had higher NLR than those in lower fibrosis stages (F1–2) (mean 2.9 vs. 1.8, *P* < 0.001). Each one-unit increase in NLR increased by 70 and 50% the likelihood of having NASH and fibrosis, respectively

Shahawy et al. (84)	NLR	90 subjects (30 with NASH, 30 with simple steatosis, and 30 healthy control)	NLR levels were significantly higher in NASH and simple steatosis groups compared to healthy controls (mean: 2.19, 1.55, and 1.19, respectively, *P* < 0.001)

Leithead et al. (72)	NLR	570 patients with end-stage cirrhosis (54 due to NAFLD) listed for liver transplantation	After adjusting for MELD, NLR ≥ 5 was associated with higher 3-month mortality (OR 6.02, *P* = 0.043). The proportion of patients who died by 3 months of listing was 3, 13.8, and 37.3% for NLR < 2, 2–4.9, and ≥5, respectively, *P* < 0.001. The listing NLR increased with increasing severity of ascites (median: 2.2, 3.1, and 4.6, for no ascites, controlled ascites, and refractory ascites, respectively, *P* < 0.001). NLR had positive correlation with listing serum bilirubin (*r* = 0.277, *P* < 0.001), listing INR (*r* = 0.156, *P* < 0.001), MELD score (*r* = 0.297, *P* < 0.001), and negative correlation with serum albumin (*r* = −0.090, *P* = 0.033), and serum sodium (*r* = −0.453, *P* < 0.001)

Yilmaz et al. (22)	NLR	102 patients (38 with NASH, 19 with HCV, and 45 with HBV) and 35 healthy controls	NLR was significantly higher in NASH patients compared to controls, HBV, and HCV patients (*P* < 0.001, *P* < 0.001, and *P* < 0.001, respectively); and was positively associated with NAFLD activity scores (*r* = 0.861, *P* < 0.001), liver fibrosis (β = 0.631, *P* < 0.001), and NASH (β = 0.753, *P* < 0.001)

Abdel-Razik et al. (71)	NLR	873 patients with biopsy-proven NAFLD (120 with NASH and 753 with simple steatosis) and 150 healthy controls	Patients with NASH had higher NLR than those without (mean: 2.6 vs. 1.9, respectively, *P* < 0.001). The NLR correlated positively with NAFLD activity score, pro-inflammatory cytokines, and CRP (*P* < 0.001). In addition, patients with advanced fibrosis stages (F3–4) had a higher NLR than those with mild (F1–2) (mean 2.5 vs.1.8, respectively, *P* < 0.001); with the highest specificity (79.2%) and sensitivity (69.4%) for identification of advanced fibrosis at NLR cutoff of 2.4 (AUC = 0.732, *P* < 0.001)

Rau et al. (30)	Th17 and the T regulatory cells	51 patients [30 with NASH and 31 with NAFLD (without histology)] and 43 healthy controls	Patients with NASH (and in less degree with steatosis) had a lower frequency of T regulatory cells in their peripheral blood, in comparison with controls. Progression from steatosis to NASH was marked by a higher frequency of Th17 cells in the liver, and an increased Th17/resting Treg ratio in the liver and in peripheral blood

*NLR, neutrophil-to-lymphocyte ratio; WBC, white blood cell*.

## Underlying Mechanisms, Pathways, and Relationship Between Cells and Cytokines in NAFLD

The understanding of the pathophysiological mechanisms linking the cellular and cytokines immune imbalances to NAFLD is still limited, and a subject of ongoing studies, as is the clarification between initiators and perpetuator imbalances. However, recent studies have been consistent in pointing out the hyperactivation of the interleukin-17 axis and TGF-β signaling pathway as the central elements in the pathogenesis of NAFLD, as well as other chronic liver diseases independently of underlying cause (15, 24, 29, 31, 48). So, the sum of the various imbalances in the immune system results in the dominance of activated pro-inflammatory pathways over the regulatory, as shown by a concurrent imbalance in Th17/Treg cells (30), culminating in the activation of the IL-17 axis.

### Neutrophils and Interleukin-6

As shown in Table 2, NLR is one of the most studied cell imbalances in recent times on outcome prediction in NAFLD. Neutrophils are the main cells of innate immunity, and its dominance is associated with the polarization to a more pro-inflammatory response, including its participation in the IL-17 activation chain, a key cytokine in organic fibrosis (85, 86). This occurs because neutrophils (and macrophages) produces the IL-6, as the main cytokine, which is in turn important in the differentiation of Th17 cells from naive T helper cells (37, 38, 87). On the other hand, a recent study showed that neutrophils are themselves an important source of IL-17 in the human liver, especially in late fibrosis stages (70). In addition to the IL-17 axis activation, the predominance of neutrophils may be associated with other mechanisms such as oxidative stress and activation/release of matrix metalloproteinases (MMPs) (88, 89).

### The Th17/Treg Differentiation and the IL-17 Axis Activation

The Th17 differentiate from the naive T helper cells in the presence of IL-6, TGF-β, IL-21, and IL-23 (37, 38, 90). Th17 cells secrete the IL-17, IL-21, and e IL-22, being important in immunity against extracellular infectious agents such as bacteria and fungi but also contribute in the immunopathogenesis of many diseases such as psoriasis and tumors (51, 91–93). IL-17 is the most studied Th17-secreted cytokine in liver disease (15, 41, 42). The differentiation of Treg, the functional counterpart of Th17 cells, has the TGF-β as a pivotal factor in the presence of retinoic acid (94–97). The main function of the Treg cells is to regulate different aspects of the immune response in order to ensure the immunologic tolerance (98, 99). The dynamic Th17/Treg balance in peripheral blood is an important element to ensure the equilibrium between the defense and the autoimmunity and is regulated by various factors, such as IL-6, IL-10, TGF-β, and the microbiome (33, 34, 90, 100). So, these cells and their precursors are interconnected and have plasticity, which causes to direct their response, in the presence of different stimuli, to different cellular type and/or cytokine profile (34–36). For example, in the presence of pro-inflammatory cytokines such as IL-6, IL-1β, and TNF-α, the normal TGF-β-driven Treg differentiation is shifted to Th17 differentiation (33, 90). So, neutrophils (and macrophages), through the production of the IL-6, participates in the IL-17 activation chain; and IL-17, in turn, is an important in granulopoiesis (101) and participates in neutrophil recruitment and organs infiltration after initial injury, and induces neutrophils cytokines and chemokines production, promoting further injury (24, 79, 102–104).

#### The Role of IL-17 Axis Activation and Associated Signaling Pathways in Fibrogenesis and Steatohepatitis

Interleukin-17 is a pro-inflammatory cytokine that is known to be produced mainly by T helper lymphocytes sub type 17 (Th17) and neutrophils, as discovered more recently, which is associated with the progression of NAFLD (15, 24, 41, 70). In the liver, the IL-17 exacerbates the liver tissue inflammation (29, 105, 106), enhancing tissue leukocytes infiltration (107), is a mediator of the cross talk between the immune system and liver cells (85, 108–111), has a profibrotic effect as noted in liver biopsies (70, 85, 86), among several others effects. In addition, is a potent stimulator of production of other inflammatory mediators, such as tumor necrosis factor (TNF-α), interleukin-1 (IL-1), and IL-6 (85, 111, 112). And by induction of IL-6 production in the hepatic cells and serum, it mediates the cross talk between liver cells, the innate, and adaptative immune responses (85, 109, 113) and has a feedback on its axis at both local and systemic level (40).

In experimental studies, the activation of the IL-17 axis showed to be central to the development of NAFLD and progression to NASH and fibrosis (15, 41, 85, 110). And the neutralization or the lack of this axis caused significant attenuation of obesity, methionine choline-deficient diet (MCDD), or schistosoma-induced liver inflammation and fibrosis (15, 41, 42, 86, 104, 114). In addition, livers of IL-17(−/−) mice were protected from NASH development (42).

One of the mechanisms by which the IL-17A exerts its profibrotic effect is using the TGF-β signaling pathways, promoting an upregulation of its receptor on hepatic stellate cells (48, 109). In addition, IL-17 inhibits the natural TGF-β-driven Treg differentiation by the pro-inflammatory environment it promotes, and by stimulating the IL-6 production, the most potent inductor of Th17 cells differentiation (38, 87, 90, 111), thereby enhancing further Th17/Treg imbalance (29, 33, 39, 115).

The relationship of the IL-17, NLR, and fibrosis has been found in other liver diseases such as viral hepatitis (116, 117), cirrhosis, and HCC (27, 28), which suggests the involvement of common points in pathogenic pathways (22, 31, 116–118). The maintenance of these imbalances seems to favor the inflammatory microenvironment, which would explain their prognostic implications, and therapeutics potentials, from NAFLD, to cirrhosis, and HCC (28, 51). In addition, the role of the IL-17 axis in fibrogenesis has been shown in organs other than the liver, including the heart (119, 120), lung (121), and kidney (122). This model has been reasonably proven by evidence of elevation of neutrophils, Th17, and related cytokines, both in the systemic circulation and in the liver (28, 29, 70). So, is this inflammatory arsenal that would act in both hepatic inflammatory infiltration and in fibrogenesis.

### Deficient Synthesis or Release of Anti-inflammatory and Antifibrotic Cytokines

**Figure 1:**
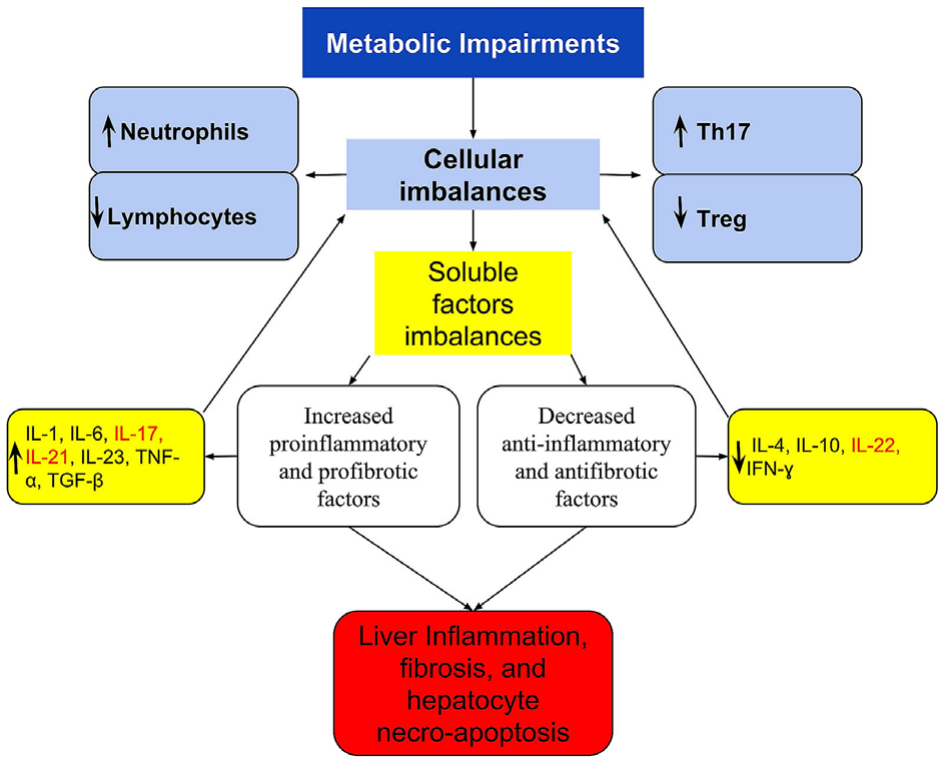
**A simplified scheme of imbalanced immune response, connecting the cellular and cytokines imbalances with the activation of IL-17 axis and the progression of NAFLD**. Metabolic disorders lead to immune imbalances in the peripheral blood and/or in the liver that are expressed at the cellular level by Th17/Treg imbalance and by the dominance of neutrophil over lymphocytes. These are reflected in imbalanced soluble factors with the dominance of pro-inflammatory and profibrotic over the anti-inflammatory and antifibrotic, which culminates in the Th17/IL-17 axis hyperactivation. In the liver, these imbalances are responsible for the recruitment and organ infiltration by neutrophil (102, 103), for increased hepatic gene expression of Th17-related cytokines (IL-17, IL-21, and IL-23) (29), resulting in steatohepatitis and fibrosis. The extra-hepatic effect, is increased production and release of neutrophils (101), and greater polarization to Th17 response, increasing further cellular imbalances, and acting as a feedback loop. Th17-secreted cytokines are listed in red.

The NAFLD immune imbalances, in addition to the above, appears to be also associated with deficient synthesis or release of anti-inflammatory and antifibrotic cytokines as IL-10 (58, 61), IL-4 (61, 123), IL-22 (42), and interferon gamma (IFN-γ) (124) that have a protective effect by suppressing the maturation of Th17 cells or counterbalancing the IL-17 effects (42, 61, 125–127). For example, Treg requires IL-10 signaling to suppress the Th17 cell-mediated inflammation (100), and this anti-inflammatory cytokine was decreased in morbidly obese patients with NAFLD (58). Figure 1 shows a simply proposed model connecting the cellular to cytokines imbalances, including the activation of the IL-17 and the progression of NAFLD.

## Emerging and Potentials Therapeutics Agents Targeting the Immune Response in NAFLD

The immune imbalances underlying the evolution of NAFLD has been explored as therapeutic targets for new drugs (or pleiotropic effects of old drugs) with the potential to slow the disease progression (114, 128).

### Targeting the IL-17 Axis and Related Signaling Pathway

#### Statins

Some of the drugs that have emerged with therapeutic potential in NAFLD are statins (129–131). Recent studies have increasingly highlighted the immunomodulatory role of statins (132–134); and shown in some diseases (other than NAFLD), its potential to interfere in the IL-17 axis, both by inhibiting the differentiation of Th17 cells, decreasing the production of IL-17 (62, 133, 135, 136), and inducing upregulation and recruitment of Treg (133, 137–139), shifting thus T cells response to Treg dominance (134, 140), which has an anti-inflammatory role and is a coordinator of immunologic tolerance (98, 99). This effect was also evident in atherosclerosis where the administration of statins was associated with the accumulation of T regulatory cells in atherosclerotic plaque (141, 142). Among the mechanisms underlying this effect on Treg induction is by modulating the TGF-β1 signal transduction (139). It is very likely that other pleiotropic effects, often unpredictable on statins, may participate in the mediation of this benefit, such as those related to the antioxidant effect (143).

In NAFLD, the use statins reduced the risk of both NASH (OR 0.57, *P* = 0.055) and fibrosis (OR 0.47, *P* = 0.011) (144). In a study involving 42 patients with dyslipidemia and biopsy-proven NASH who underwent treatment with atorvastatin (10 mg/day) for 12 months, atorvastatin improved NASH activity score and increased liver to spleen density ratio, and this improvement was accompanied by a significant reduction of inflammation markers (145). In parallel, atorvastatin significantly decreased liver transaminase, γ-glutamyl transpeptidase, low-density lipoprotein cholesterol, and triglycerides (145). In another study with 20 patients with HIV and biopsy-proven NASH, the use of rosuvastatin ameliorated NASH in 19 of 20 patients within 12 months (130). Given this known plausibility of immune imbalances in NAFLD pathophysiology and the effect of statins in reestablishing the balance, studies with appropriate design are needed to confirm or refute this effect.

#### Vitamin D

Another agent that has shown important participation on Th17/Treg lymphocytes differentiation and IL-17 axis modulation is vitamin D and its isoforms (146–148). In the liver, studies have shown a significant association between vitamin D deficiency or insufficiency with NAFLD, principally in men and diabetics patients (149–152), and increased significantly the risk of NASH, fibrosis, and NASH severity in both adults and children with NAFLD (5, 153). Vitamin D supplementation in subjects with NAFLD reduced liver fibrosis through counteracting TGF-β-induced fibrogenesis (154, 155) and reduced the inflammatory response and insulin resistance as surrogate outcomes (155, 156). In one of these studies, patients with NASH had higher levels of TGF-β1 than those with simple steatosis, and the improvement of the inflammation and fibrosis after treatment was accompanied by a reduction in TGF-β1 levels (155). In the experimental model, the supplementation slowed the development and progression of NASH (157). So, available vitamin D compounds or vitamin D receptor agonists can bring another target to treat NAFLD, either as by modulating the IL-17 axis or other mechanisms.

#### Monoclonal Antibodies

It is worth remembering that there are already monoclonal antibodies against IL-17 (secukinumab and ixekizumab), already released for the treatment of rheumatic diseases that have activation of IL-17-axis as a crucial point in its pathogenesis (91), and the newest and attractive tregalizumab a monoclonal antibody that binds to CD4 T cells and induces Treg activation (158). Considering the key role of IL-17 in NAFLD, these agents may have a protective effect on the progression of NAFLD. In fact, in experimental studies, anti-IL-17 antibody improved hepatic steatosis by suppressing interleukin-17-related fatty acid metabolism (159). However, there are no clinical studies that have tested the use of this agent in NAFLD.

#### Probiotics and Retinoic Acid

Another aspect that opens new therapeutic potentials is the consideration of the intestine as a primary source of lymphocytes in NAFLD, and the modulator role of microbiota in this cellular population (64, 65). In fact, the administration of lactobacillus and other probiotics shows to decrease Th17 cell population and IL-17 secretion, while increasing Treg cell population (160–162). In addition, it has been shown that retinoic acid has an important role as a modulator of the cell response differentiation in gut, favoring to the Treg cells (94, 95, 163, 164). So studies with retinoic acid or its receptor agonists may bring the next NAFLD treatments targeting the IL-17 axis. It wins a particular interest by the observation that low levels of retinoic acid in serum or its receptor in hepatic tissue are associated with higher severity of NAFLD (165); and the demonstration that the administration of all-trans retinoic acid ameliorates hepatic steatosis in experimental research (166). Like statins, retinoic acid modulates the TGF-β1 signal transduction inducing to Treg response, and this may be one of the mechanisms underlying its beneficial effect (95, 97).

#### Other Potential Agents

Several other agents have the potential to act on the IL-17 axis (shifting the Th17/Treg balance in favor of Treg dominance), including agents such as rennin–angiotensin system blockers and digoxin (51, 142, 167). In a study including 159 hypertensive patients, the association of telmisartan with rosuvastatin showed a synergistic effect on ameliorating Th17/Treg functional imbalance, with a significant decrease in Th17 cells frequency, IL-17, IL-6, TNF-α, IL-1β, IL-2, IFN-γ, hs-CRP, and MCP-1, TGF-β3 (142). In another study, animals treated with digoxin, which also inhibits Th17 differentiation, presented with reduced levels of circulating Th17 cells and serum IL-17A, associated with reduced liver steatosis, liver immune cell infiltration, and liver injury; and increased glucose tolerance and insulin sensitivity than non-treated mice (51). Other substances able to shift the imbalance of Treg/Th17 cells to Treg dominance and that have shown to relieve NAFLD are 3, 3′-diindolylmethane and flavonoids (32, 61).

## Conclusion and Future Directions

The available studies point to an important value of immunes imbalances, either at cellular or cytokines levels, in the pathogenesis of NAFLD. Particularly, the imbalances between neutrophils and lymphocytes counts (NLR) and at T helper cellular subsets (expressed by Th17/Treg imbalance). The reflection of this at the molecular level is a pro-inflammatory environment that includes IL-6, TNF-α, and TGF-β, and culminates in the hyperactivation of the IL-17 axis. The knowledge of this participation can help better understand the pathogenesis, offer non-invasive tools to evaluate the disease, and support the development of new therapeutic targets. Among these targets, the IL-17 axis and related signaling pathway is a potential; and agents such as statins, vitamin D, retinoic acid, probiotics, and monoclonal antibodies against IL-17 have a promissory perspective.

New studies should be designed for clarification between initiators, and perpetuator imbalances, and to prove the overall clinical utility of these imbalances as potential disease biomarkers. The effectiveness of therapies targeting the various mediators and pathways involved in disease pathogenesis should be evaluated by well-designed clinical randomized trials with adequate sample size and with histological assessment of the disease.

## Author Contributions

FP: prepared the manuscript text and figures.

## Conflict of Interest Statement

The author declares that the research was conducted in the absence of any commercial or financial relationships that could be construed as a potential conflict of interest. The reviewer JW and handling Editor declared their shared affiliation, and the handling Editor states that the process nevertheless met the standards of a fair and objective review.
